# Mycotoxin Contamination in the EU Feed Supply Chain: A Focus on Cereal Byproducts

**DOI:** 10.3390/toxins8020045

**Published:** 2016-02-15

**Authors:** Luciano Pinotti, Matteo Ottoboni, Carlotta Giromini, Vittorio Dell’Orto, Federica Cheli

**Affiliations:** Department of Health, Animal Science and Food Safety, Università degli Studi di Milano, Via Trentacoste, 2, 20134 Milan, Italy; matteo.ottoboni@unimi.it (M.O.); carlotta.giromini@unimi.it (C.G.); vittorio.dellorto@unimi.it (V.D.); federica.cheli@unimi.it (F.C.)

**Keywords:** mycotoxins, feed, food processing, milling byproducts, DDGS, brewery byproducts, analysis

## Abstract

Mycotoxins represent a risk to the feed supply chain with an impact on economies and international trade. A high percentage of feed samples have been reported to be contaminated with more than one mycotoxin. In most cases, the concentrations were low enough to ensure compliance with the European Union (EU) guidance values or maximum admitted levels. However, mycotoxin co-contamination might still exert adverse effects on animals due to additive/synergistic interactions. Studies on the fate of mycotoxins during cereal processing, such as milling, production of ethanol fuels, and beer brewing, have shown that mycotoxins are concentrated into fractions that are commonly used as animal feed. Published data show a high variability in mycotoxin repartitioning, mainly due to the type of mycotoxins, the level and extent of fungal contamination, and a failure to understand the complexity of food processing technologies. Precise knowledge of mycotoxin repartitioning during technological processes is critical and may provide a sound technical basis for feed managers to conform to legislation requirements and reduce the risk of severe adverse market and trade repercussions. Regular, economical and straightforward feed testing is critical to reach a quick and accurate diagnosis of feed quality. The use of rapid methods represents a future challenge.

## 1. Introduction

The feed supply chain is a crucial element for all livestock production systems. According to the FEFAC [1], within the European Union-28 (EU-28), approximately 475 million tons of feedstuffs and forages are consumed by livestock each year. In 2013, 153 million tons of compound feed were produced by EU compounders, accounting for 80% of all of the purchased feedstuffs. Feed supply and feed safety are intimately linked; the origin of feedstuffs, processing, handling and storage, as well as many other factors related to the market, can affect at different levels both the quality and safety of feed [2]. Among the most important safety risks for the feed industry and the security of the feed supply chain are mycotoxins. Globally, mycotoxins have a significant impact on human and animal health, economies and international trade [3,4,5,6,7]. Despite efforts to control fungal contamination, extensive mycotoxin contamination has been reported to occur in feed and food. Mycotoxin contamination of feed is an area of great concern because of the negative health effects on animals. Furthermore, according to the possible carry-over of each toxin, feed contamination can also represent a hazard for the safety of food of animal origin and contribute to mycotoxin intake in humans [8]. Recent surveys have been carried out to evaluate the incidence of mycotoxin contamination. On a global level, 30% to 100% of food and feed samples are co-contaminated [9,10,11,12,13]. Therefore, when we consider the exposure of animals to mycotoxins, co-contamination is of particular concern because of the detrimental additive and/or synergic effects of mycotoxins on animal health.

Cereals and cereal byproducts constitute a major part of the daily diet of animals and are important ingredients in animal compound feed. Average inclusion rates of 48% and 11.5% of cereals and co-products of the food and bioethanol industry, respectively, have been reported [14]. Food processing affects mycotoxin distribution and concentration. Cereal processes concentrate mycotoxins into fractions that are commonly used as animal feed [15,16,17]. Therefore, the mycotoxin distribution in cereal processing procedures is a worldwide topic of interest due to the high economic and health impacts of mycotoxins and is an important tool in risk management to establish limits for raw commodities to ensure safe food byproducts for feed use.

The feed industry is a sustainable outlet for food processing industries, converting byproducts into high-quality animal feed. Mycotoxin occurrence in grain and grain co- and byproducts from different technological processes is a worldwide topic of interest for the feed industry in order to increase the marketability and acceptance of these products as feed ingredients and include them safely in the feed supply chain. This paper reviews the most recent findings on feed mycotoxin contamination and the effects of the cereal technological processes on mycotoxin distribution in products and byproducts. In addition, the main interventions and effective tools to properly manage the mycotoxin risk at industrial level are discussed.

## 2. Global Occurrence of Mycotoxins in Feed

Despite efforts to control fungal contamination, extensive mycotoxin contamination has been reported in both developing and developed countries. The knowledge of mycotoxin occurrence in animal feed is concentrated primarily on commodities and feedstuffs. However, in animal feeding, the contribution of forages to total mycotoxin intake could be significant and sometimes greater than that of compound feed in the ruminant diet, as forages are the main dry matter component [18]. In this respect, a survey conducted by Driehuis *et al.* [19] estimated the total dietary intake of mycotoxins by dairy cows on 24 farms in the Netherlands. Silage (mixture of grass and corn silage) and compound feed were the main components of the diet, representing an average 67% and 23% of the dry matter intake, respectively. The authors found that, relative to compound feed, the contribution of silage to total intake of deoxynivalenol (DON) and zearalenone (ZEA) was 3.5 and 2.9 times greater, respectively. Furthermore, other recent studies reported not only that preserved forages, like silage [20], can be an important source of mycotoxins in ruminant diet, but also that fresh forage and/or pasture can be a route of exposure to these contaminants [21]. Thousands of potential toxic metabolites of fungi have been reported [22]. However, for practical consideration in feed manufacturing, because of their worldwide occurrence and concern regarding human and animal diseases, the number is considerably less [9].

**Figure 1:**
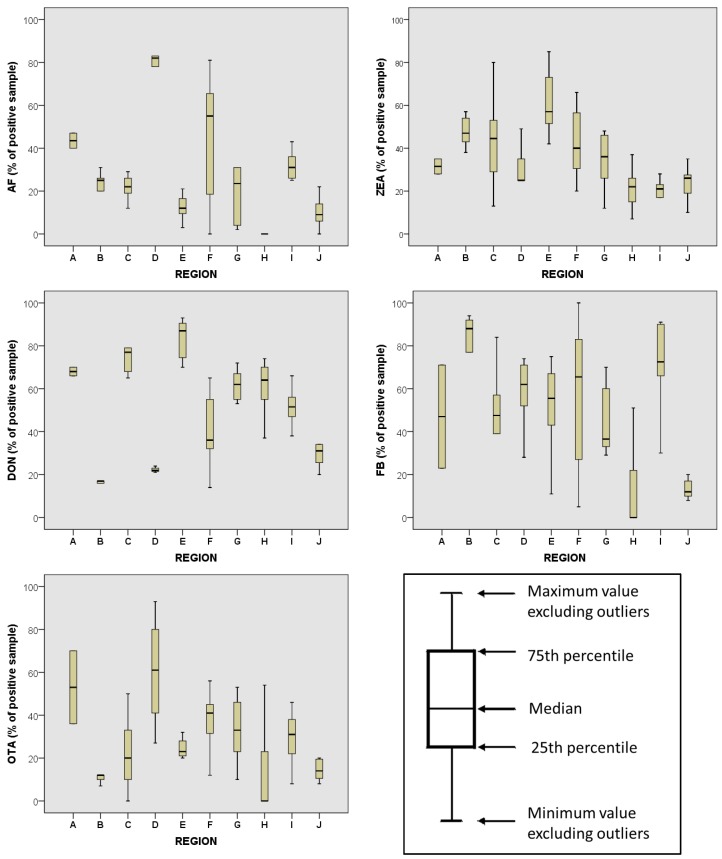
Worldwide mycotoxin occurrence (percentage of mycotoxin contaminated samples) in raw feed materials and finished feed (data from [9,10,11,12,13,23,24]). A = Africa; B = South America; C = North America; D = South Asia; E = North Asia; F = Southeast Asia; G = Central Europe; H = North Europe; I = South Europe; J = Oceania.

Recent surveys were carried out to evaluate the worldwide incidence of mycotoxin contamination in feed and feed raw materials, mainly grains and grain co-products (bran, corn gluten meal, dried distillers’ grains and solubles) and, to a lesser extent, other feed ingredients (e.g., soybean meal, cotton seed, sorghum, cassava, peanut, copra, *etc.*; 12% of the total analyzed samples) [9,10,11,12,13,23,24]. The overall results confirm that aflatoxins (AFs), DON, fumonisins, ochratoxin A (OTA), T-2 toxin and ZEA are the main contaminating mycotoxins in feed. Considerable differences regarding the type and prevalence of mycotoxin contamination in different regions of the world have been reported (Figure 1).

AFs are most often detected in Southern Europe, Africa, South Asia and Southeast Asia (average values of positive samples higher than 30%). The highest incidence of DON contamination (more than 60% of positive samples) was found in samples from North America, Northern and Central Europe, Africa and North Asia. The highest incidence of ZEA contamination (more than 30% of positive samples) was found in North and South America, Central Europe, Africa, and North and Southeast Asia. The highest incidence of fumonisin contamination (more than 50% of positive samples) was found in South America, Southern Europe, Africa, North, and South and Southeast Asia. OTA prevalence was highest in South Asia and Africa (more than 50% of positive samples). Range values of various mycotoxins in feed material and feedstuffs, surveyed worldwide by Marquardt and Madhyastha [25], are reported in Table 1.

**Table 1:** Mycotoxins (ppb) in feed material and feedstuffs surveyed worldwide (adapted from [25]).

Geographical Region	AFB1	ZEA	DON	FB1	OTA
North America	8	271	1,947	902	1
Central South America	2–3	0–111	51–237	1030–3121	0–9
Europe	0–3	3–37	88–968	925–3052	0–9
Asia	8–90	32–219	61–691	380–797	1–15
Oceania	1	50	94	109	1
Africa	42	25	745	855	6

Differences in mycotoxin occurrence and concentration between distant geographical areas are uncontroversial. However, within each geographical area, seasonal and local weather conditions during critical plant growing stages (before, during flowering or in grain at maturity) are of great importance to explain the variation in the results reported by the surveys. Therefore, the high variability in the occurrence of mycotoxins may be the results of several factors: the different type of the analyzed samples, the years of the surveys, and the annual weather fluctuations. Mycotoxin contamination by emerging *Fusarium* mycotoxins, such as beauvericin and enniatins represents a problem of concern especially in Northern Europe [26,27,28,29]. However, there is evidence of the presence of beauvericin in feed ingredients and samples of compound feeds from East Asian countries [30]. This global scenario confirms that contamination is strongly dependent on regional climatic conditions. In general, environmental conditions, such as excessive moisture, temperature extremes, humidity, drought conditions, insect damage, crop systems and some agronomic practices can cause stress and predispose plants in the field to mold and determine the severity of mycotoxin contamination [4,31,32,33]. The main climatic conditions that lead to aflatoxin accumulation are high temperature, low rainfall and severe drought stress. *Fusarium* spp. producing DON and ZEA are generally associated with a cool and excessively wet growing season [32,34]. Despite progress made in prevention through breeding of resistant varieties and improvement in agronomic practices [32], hazardous concentrations of mycotoxins may occur as a result of annual weather fluctuations. A further scenario is represented by the climate changes. In general, as reported by Paterson and Lima [35], if the temperature increases in cool or temperate climates, the relevant countries may become more liable to aflatoxins. Tropical countries may become too inhospitable for conventional fungal growth and mycotoxin production. By contrast, cold regions may become liable to temperate problems concerning OTA, patulin and *Fusarium* toxins (e.g., DON), opening new challenges for both feed and food sector. Although mycotoxins were ubiquitously present in the analyzed feed and feed materials in the different surveys, the levels of detected mycotoxins were generally low. Schatzmayr and Streit [24] found that only 17% of the aflatoxin-tested samples, coming from different world regions, did not comply with the most stringent EU maximum level of 5 μg/kg aflatoxin B_1_ applicable to feed for dairy animals [36]. Concentrations above the EU guidance levels for ZEA, DON, fumonisins and OTA were detected in 17%, 15%, 3.2% and 0.9% of the samples, respectively, an important topic raised from several reviews on mycotoxin occurrence in feed materials [9,10,11,12,13,20,24,37]. Multi-mycotoxin studies reported a high incidence of 30% to 100% of analyzed samples that were contaminated with two or more mycotoxins [11,12,29]. The authors conclude that several factors may be responsible for mycotoxin co-occurrence, as most fungi are able to simultaneously produce several mycotoxins, and commodities can be contaminated by several fungi. Compound feed, as a mixture of several ingredients, may be particularly vulnerable to multiple mycotoxin contamination. Moreover, globalized feed grain trade may distribute mycotoxins outside of their natural occurrence geographical areas, complicating the prediction of mycotoxin contamination in compound feed. The co-occurrence of mycotoxins has been evaluated in compound feed from different regions [11]. Multi-mycotoxin contamination was more prevalent in samples from Asia (82%) than in samples from Europe and America (40%). Multi-mycotoxin contamination is a topic of great concern, as co-contaminated samples might still exert adverse effects on animals due to additive/synergistic interactions of the mycotoxins. The complexity of mycotoxin interactions varies according to the animal species, the level and type of mycotoxin contamination and the length of exposure [38,39,40,41,42,43].

In terms of mycotoxin contamination, particular attention must be given to the modified mycotoxins, which represent an emerging issue for food and feed safety [44]. Plants are capable of transforming mycotoxins into conjugated forms, reducing the toxicity of mycotoxins [45]. Plant metabolites have been identified so far for DON, nivalenol (NIV), fusarenon-X, T-2 toxin, HT-2 toxin, ZEN, OTA, destruxins, fusaric acid and modified fumonisins have been found especially in cereal commodities, such as wheat, corn, and barley [45,46,47,48,49]. Toxicological data on modified mycotoxins, including those of processing origin, are still limited. Glucoside conjugates of trichothecenes may represent a potential safety threat because they can be hydrolyzed to toxic compounds during mammalian digestion [45,50]. However, recent advances in modified mycotoxin occurrence and toxicity, such as DON-3G, have also suggested that mycotoxins conjugates have a lower toxicity potential, due to the lower absorption in the gastrointestinal tract [51,52,53].

## 3. Mycotoxins in Feed: Economic and Legislative Context

Aside from health risks, important economic and trade implications arise from the mycotoxin contamination of feed [3,6,7]. The health and economic impact of mycotoxins, considering the seasonality of contamination for the different toxins, includes loss of crop production, disposal of contaminated food and feed, reduced livestock production, loss of human and animal life, increased human and animal health care costs, analytical and regulatory costs, and investment in research. The economic costs and impact on the international trade associated with mycotoxin contamination are difficult to assess. Quantitative estimates of economic losses associated with mycotoxin contamination in commodities may range from hundreds of millions to billions of US$ annually [3,6,7]. The globalization of the trade in agricultural commodities and the lack of legislative harmonization have contributed significantly to the discussion about the awareness of mycotoxins entering the feed/food supply chain. Mycotoxin regulations have been established in more than 100 countries [54,55], and the maximum acceptable limits vary greatly from country to country. The European Union harmonized regulations for the maximum levels of mycotoxins in food and feed among its member nations (Table 2) [56,57]. Maximum levels and guidance values for mycotoxins in animal feed have been set in Commission Directive 2003/100/EC [36] and Commission Recommendation 2006/576/EC [58] (Table 3). In the case of lots intended for industrial purposes (e.g., bioethanol or biopolymer production), neither maximum limits nor guidance levels have been established. Another important topic is the presence of modified mycotoxins in feed and food. The European legislation must consider these topics and include both modified forms and emerging mycotoxins in the near future, as also recommended by two EFSA Scientific opinions [44,59].

**Table 2:** Maximum levels for mycotoxins in cereals and cereal products for human consumption ([60,61]).

Mycotoxin	Cereal and Cereal Products	Maximum Levels, µg/kg
Aflatoxin B_1_	All cereals and all products derived from cereals	2.0
	Maize to be subjected to sorting or other physical treatment before human consumption or use as an ingredient in foodstuffs	5.0
Aflatoxins, sum of B_1_, B_2_, G_1_ and G_2_	All cereals and all products derived from cereals	4.0
	Maize to be subjected to sorting or other physical treatment before human consumption or use as an ingredient in foodstuffs	10.0
Deoxynivalenol	Unprocessed cereals other than durum wheat, oats and maize	1250
	Unprocessed durum wheat and oats	1750
	Unprocessed maize, with the exception of unprocessed maize intended to be processed by wet milling	1750
	Cereals intended for direct human consumption, cereal flour, bran and germ as end product marketed for direct human consumption	750
Zearalenone	Unprocessed cereals other than maize	100
	Unprocessed maize with the exception of unprocessed maize intended to be processed by wet milling	350
	Cereals intended for direct human consumption, cereal flour, bran and germ as end product marketed for direct human consumption	75
	Maize intended for direct human consumption, maize-based snacks and maize-based breakfast cereals	100
Ochratoxin A	Unprocessed cereals	5.0
	All products derived from unprocessed cereals, including processed cereal products and cereals intended for direct human consumption	3.0
Fumonisin B_1_ + B_2_	Unprocessed maize, with the exception of unprocessed maize intended to be processed by wet milling	4000
	Maize intended for direct human consumption, maize-based foods for direct human consumption	1000
Sum T-2 and HT-2 toxin(*)	Unprocessed cereals	-
	Barley and maize	200
	Oats	1000
	Wheat, rye and other cereals	100
Sum T-2 and HT-2 toxin(*)	Cereals grains for direct human consumption	-
	Oats	200
	Maize	100
	Other cereals	50

* Indicates recommendations.

**Table 3:** Maximum levels and guidance levels for mycotoxins in products intended for animal feed ([36,58,61]).

Mycotoxin	Cereal and Cereal Products	Maximum Levels, mg/kg
Aflatoxin B_1_(*)	All feed materials	0.02
	Complete feedstuffs for cattle, sheep and goats with the exception of:	0.02
	Complete feedstuffs for dairy animals	0.005
	Complete feedstuffs for calves and lambs	0.01
	Complete feedstuffs for pigs and poultry (except young animals)	0.02
	Other complete feedstuffs	0.01
	Complementary feedstuffs for cattle, sheep and goats (except Complementary feedstuffs for dairy animals, calves and lambs)	0.02
	Complementary feedstuffs for pigs and poultry (except young animals)	-
	Other complementary feedstuffs	0.02
	Complete feedstuffs for cattle, sheep and goats with the exception of:	0.005
Deoxynivalenol	Feed materials	-
	Cereals and cereal products with the exception of maize byproducts	8
	Maize byproducts	12
	Complementary and complete feedstuffs with the exception of:	5
	Complementary and complete feedstuffs for pigs	0.9
	Complementary and complete feedstuffs for calves (<4 months), lambs and kids	2
Zearalenone	Feed materials	-
	Cereals and cereal products with the exception of maize byproducts	2
	Maize byproducts	3
	Complementary and complete feedstuffs	-
	Complementary and complete feedstuffs for piglets and gilts (young sows)	0.1
	Complementary and complete feedstuffs for sows and fattening pigs	0.25
	Complementary and complete feedstuffs for calves, dairy cattle, sheep (including lambs) and goats (including kids)	0.5
Ochratoxin A	Feed materials	-
	Cereals and cereal products	0.25
	Complementary and complete feedstuffs	-
	Complementary and complete feedstuffs for pigs	0.05
	Complementary and complete feedstuffs for poultry	0.1
Fumonisin B_1_ + B_2_	Feed materials	-
	Maize and maize products	60
	Complementary and complete feedstuffs	-
	Pigs, horses (Equidae), rabbits and pet animals	5
	fish	10
	Poultry, calves (<4 months), lambs and kids	20
	adult ruminants (>4 months) and mink	50
Sum T-2 and HT-2 toxin	Cereal products for feed and complementary feed	-
	Oat milling products	2000
	Other cereal products	500

* Indicates Maximum levels.

As a concrete result of the European integration, in terms of ensuring the highest possible level of the safety of the food chain and compliances with EU food and feed legislation, The Rapid Alert System for Food and Feed (RASFF) was launched in 1979. RASFF is a tool to exchange information between competent authorities on consignments of food and feed in cases in which a risk to human and animal health has been identified and measures have been taken. According to the annual reports of the RASFF, mycotoxins still represent an important hazard category, although the number of notifications has decreased throughout the years. According to the annual report of the RASFF, in 2013 [62], out of the 3137 original notifications that were transmitted, 237 concerned feed, with mycotoxins representing the second hazard category with 37 notifications. In 2014 (preliminary report), out of the 3157 original notifications that were transmitted through the RASFF, 309 concerned feed materials and 26 concerned mycotoxins in feed [63]. Notifications concerning feed mycotoxins have decreased since 2011 but still represent the third-most-important hazard category for feed.

## 4. Cereals and Cereal Byproducts as Animal Feed

**Figure 2:**
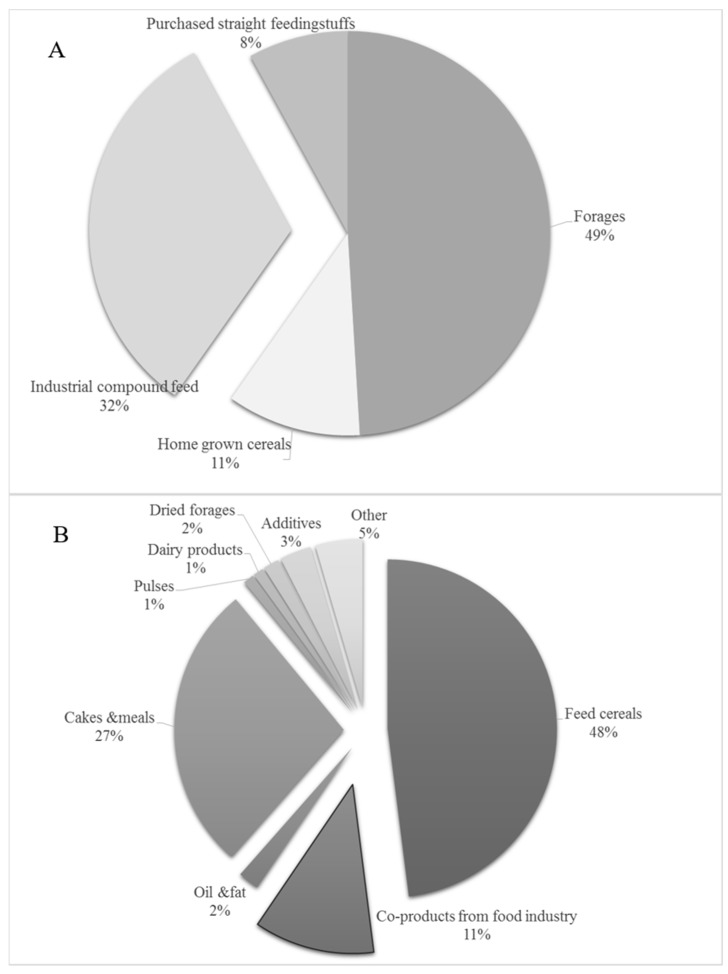
Feedstuffs categories (**A**) and Compound feed ingredients (**B**) consumed and used, respectively, by the EU-28 livestock sector in 2014 (Modified by [14]).

Cereals and cereal byproducts constitute a major part of the daily diet of the human and animal populations. FAO’s latest forecast for global cereal production in 2015 stands at 2540 million tons [64]. For the feed sector, cereals represent the main components of industrial feeds, whose estimated production worldwide is more than 900 million tons [65]. The estimates of the European association of cereals, rice, feedstuffs, oilseeds, olive oil, oils and fats and agrosupply trade (COCERAL) for the EU-28 cereal production in 2014 was 323.3 million tons [66]. In the EU-28 in 2014–2015, the cereal market share was 26% for the feed industry, 34% for on-farm feed use, 23% for food/human consumption, 4% for biofuel production, 2% for seed production, and 8% for other internal use [14]. To sustain the European livestock production, about 475 million tons of feedstuffs are required each year within the EU-28. The main feedstuffs categories and compound feed ingredients consumed and used, respectively, by the EU-28 livestock sector are reported in Figure 2. Cereals are the main ingredients in farm animal diets, but also the co- and byproducts obtained from them are an important category in animal compound feed formulas [67]. Byproducts consumption by the feed industry in the EU-28 is about 20 million tons per year [14].

Cereals processing affects mycotoxin distribution and concentration. Therefore, knowledge of the mycotoxin distribution factor in cereal processing procedures is a worldwide topic of interest for the feed industry to increase the marketability and acceptance of these products as feed ingredients.

## 5. Fate of Mycotoxins during Cereal Processing

Cereal grains may become contaminated by molds and mycotoxins while in the field and during storage. The mycotoxins commonly occurring in cereal grains are not destroyed during most food processing operations. However, food processing affects mycotoxins distribution and concentration. Several food processes concentrate mycotoxins into fractions that are commonly used as animal feed with potential to become residues in animal products and still enter the human food supply chain.

### 5.1. Cereal Milling

In addition to health risks, mycotoxin contamination has a detrimental effect on the quality and the processing performance of cereals. *Fusarium* damage may reduce wheat milling performance, affect flour yield and flour ash, and have a strong negative effect on flour brightness and baking performance [68]. The fate of mycotoxins during cereal processing, such as sorting, cleaning, milling and thermal processes, has been studied by several authors [15,69,70,71,72,73,74,75]. Published data confirm that milling reduces mycotoxin concentration in fractions that are used for human consumption but concentrates mycotoxins into fractions that are commonly used as animal feed. However, their level in feedstuffs is variable and affected by several factors such as the type of mycotoxins, the level and extent of fungal contamination, and the complexity of the cereal processing technology. Moreover, part of these byproducts may also represent promising novel food ingredients with a high value for human nutrition as well [76].

The effects of dry milling procedures on mycotoxin distribution in wheat fractions have been recently reviewed [15,75]. At the industrial level, the dry milling process of wheat is a gradual reduction process by which wheat is ground into flour or semolina. It includes several steps, such as cleaning and sorting, debranning and milling. Physical and mechanical processes, such as sorting and cleaning prior to milling, reduce mycotoxin contamination in wheat by removing kernels with extensive mold growth, broken kernels, fine materials, and dust. The results indicate that the effect of pre-milling processes and the efficiency of mycotoxin removal are extremely variable. The concentration of mycotoxins in cleaned wheat ranges from 7% to 63% for DON, from 7 to almost 100% for NIV, and from 7% to 40% for ZEA, of the contamination level in unclean grains [77,78,79,80]. A reduction of 62% and 53% of T-2 and HT-2, respectively, has been reported in wheat grains after cleaning [81]. Several factors may be involved in this response, such as the initial condition of the grains, the type and extent of the contamination, and the type and efficiency of the cleaning process. Cleaning can be performed according to different properties of wheat kernels: shape, size, relative density, and air resistance. Grains heavily infected with *Fusarium* become shriveled and may have a lower relative density than do healthy grains. Therefore, these grains can be removed more efficiently using gravity separators rather than by other technological approaches [82]. The debranning of wheat, a mechanical process by which the outer layers of wheat grains are removed prior to the milling process, is used in industrial processing because it can enhance the milling performance of wheat and the degree of refinement of flour and semolina [83]. Debranning before milling further reduces the level of mycotoxin content in wheat grain. As for the cleaning and sorting procedures, the effect of debranning and the efficiency of mycotoxin removal are extremely variable. A reduction of DON in debranned wheat ranging from 15% to 78% has been reported [77,84,85,86]. To the best of our knowledge, the effect of debranning on mycotoxin content in wheat is limited to DON repartition. At the laboratory scale, the efficiency of debranning is irrespective of the initial level of mycotoxins in grains, depending mainly on the length of the pearling process and the percentage of grain tissue removal [85]. More studies are needed to optimize debranning technical conditions at the industrial level to reduce mycotoxin contamination with less grain tissue removal. Albeit the high variability in removal efficiency of mycotoxin, the results indicate that the physical processes that are carried out before milling (such as sorting, cleaning, and debranning) are interesting and very efficient methods to reduce the wheat mycotoxin content before milling. As in cleaning and debranning, in the milling process there is no step that destroys mycotoxins; however, mycotoxin contamination may be redistributed in milling fractions [87,88,89]. These results indicate that the concentration of mycotoxins in fractions that are mainly intended for animal feeds (bran, flour shorts screenings and middlings) compared to that in wheat grain may be up to 800% but more typically ranges from 150% to 340% [15]. These results have been confirmed more recently by Tibola *et al.* [88], who reported an average distribution factor of 130% and 190% for DON and ZEA in bran, respectively. Few data are available regarding the distribution of modified mycotoxins in milling fractions. In wheat bran, the levels of DON-3G are 1.5-2.5-fold higher than in wheat samples [48,88,90]. In contrast, the ZEA and ZEA-14S contents are significantly higher in the fiber-rich fractions [90]. In the case of other mycotoxins, Vaclavikova *et al.* [91] reported average values of Enniatin B and Enniatin B1 three-fold and five-fold higher in bran and shorts than in wheat samples, respectively. Combining the data reported above regarding the concentration of different mycotoxins in cereal byproducts relative to unprocessed cereals, it can be speculated that, in general, the different steps in the milling process produce an increase in mycotoxin content by 1.5 to 8 times the initial level in unprocessed materials. The high variability in mycotoxin concentration in cereal byproducts may have a different impact on their safe use in feed formulation. To evaluate this impact, we can simulate different scenarios. If we consider, for example, a level of DON contamination in maize or durum wheat of 1250 and 1750 µg/kg, respectively, which are the EU limits for food, a 1.5-fold increase in DON content in byproducts will results in a DON concentration in maize and wheat byproducts of 1875 and 2625 µg/kg, respectively. In a worse scenario, an eight-fold increase in DON content in byproducts will results in a DON concentration in maize and wheat byproducts of 10,000 and 14,000 µg/kg, respectively. In the first example, the contamination levels of cereal byproducts are below the EU recommended values for maize and cereal byproducts intended for animal nutrition, while in the second example they are well above the recommended values. Regarding the application of guidance values for cereals and cereal products, it must be underlined that the Commission Recommendation 2006/576/EC recommends: “In applying these guidance values, Member States should take into account the fact that the guidance values for cereals and cereal products have been determined for the most tolerant animal species and are therefore to be considered as upper guidance values. For feed for more sensitive animals, Member States should ensure that lower guidance values for cereals and cereal products are applied by feed manufacturers taking into account the sensitivity of the animal species and enabling compliance with the guidance values determined for compound feedingstuffs for these animal species” [58]. Therefore, this imposes a case-by-case evaluation to safely manage cereal byproducts that implies some specific interventions as reported before (analysis, inclusion level evaluation, species sensitivity and use of feed additives).

The different ways of milling corn, such as dry-milling and wet-milling, are able to improve the final quality of food products by reducing mycotoxin concentrations. The dry milling of corn, removing the outer parts of the corn kernel, such as hull and bran, allows obtaining several main products, including grits, germ, meals and flours [92]. The industrial process based on a dry-milling technology is commonly coupled with a dry or wet degermination. Corn mills use comprehensive cleaning regimes, such as mechanical shelling and dehulling methods, to remove stones, metal objects, and other such contaminants, as well as dust, straw, corn cobs, and broken corn seeds. Because mycotoxins are often concentrated in the latter impurities, the cleaning step reduces the overall mycotoxin concentrations, although the extent to which this occurs in corn can vary [72,93,94,95]. However, the waste produced during the cleaning step may be integrated to the corn meal for feed. The knowledge of mycotoxin repartitioning in corn milling fractions is largely associated with aflatoxins and *Fusarium* toxins, which contaminate corn and corn-based food and feeds worldwide [9,10]. The dry milling of corn led to a heterogeneous distribution of mycotoxins in the different parts of the grain, with increased levels in fractions from outer layers and decreased levels in fractions from inner layers. Mycotoxins tend to be concentrated in corn germ, bran fractions and animal feed flour [72,95,96,97,98,99,100,101]. However, a variability of the reported distribution factors is very high. A concentration of aflatoxins in corn meal for feed from the industrial dry-milling process of 356% and 288% compared to that in corn grain has been reported [93,97]. The concentration of fumonisin B_1_ in corn meal for feed, compared to that in the grain, ranges from 200% to 350% [96,97,101]. Burger *et al.* [102] have confirmed this distribution pattern for fumonisins under experimental conditions. The study suggests that, although experimental dry milling under laboratory conditions cannot duplicate industrial milling, it provides an opportunity to better separate the different corn milling processes and investigate the fate of mycotoxins in the different milling fractions on an amendable laboratory scale. The wet milling process used for maize results in the production of food grade fractions such as maize starch and glucose syrups. The wet milling of corn resulted in the concentration of mycotoxins, including aflatoxin, ZEA, T-2 toxin and fumonisins in steep water, gluten fiber and germ, while the starch tends to be relatively free of these mycotoxins [82].

Factors that cause the variability of mycotoxin repartitioning in cereal milling fractions have not been completely determined. The high mycotoxin repartitioning in byproducts may indicate a concentration of toxins in the outer part of the kernel [79]. Peripheral tissues are the parts of the grain that are first colonized by fungi and are often contaminated by microorganisms [76,93]. However, the mycotoxin contamination of milling byproducts may not simply be due to the presence of peripheral grain tissues. When ash, phytic acid, and crude fiber were used as markers to monitor the presence of external tissues in wheat fractions, even if the highest concentration of DON was found in fractions originating from the grain outer layers, a lack of correlation was found with ash and phytic acid and a low positive correlation with fiber [77]. The cultivar effect, the degree and time of fungal infection, the weather conditions and the milling technology represent other sources of differences in mycotoxin distribution in cereal milling fractions.

A further aspect that has to be considered is the level of inclusion of these materials in farm animal diets and formulas. As reported above, byproducts represent about 11.5% of the compound feed ingredients. Their incidence in different farm animal diets can vary according to the appropriate diet formulation, which affect the potential mycotoxin exposure to the animal that can be more or less susceptible. In comparison to other animals, for instance, poultry species tend to be resistant to the effects of fumonisin, DON and ZEA. By contrast, pigs are the most sensitive species to DON as well as to T-2.

In conclusion, the results indicate that no step in the milling processes destroys mycotoxins; however, mycotoxin contamination may be redistributed in the milling fractions. The published data show a high variability in mycotoxin repartitioning that is sometimes conflicting, but this result may be mainly due to the type of mycotoxins, the level and extent of fungal contamination, and a failure to understand the complexity of the milling technology. Combining all these factors, results from the literature indicate that sometimes the limits that are proposed for cereal-derived products may be not warranted by the limit for unprocessed cereals. Therefore, the knowledge of the mycotoxin distribution factor in milling procedures is a worldwide topic of interest due to the high economic and health impact of mycotoxins and an important tool in risk management to establish limits for raw commodities to ensure safe food byproducts for feed use. The characterization and manipulation of kernel characteristics and milling practices therefore can become important strategies to further reduce mycotoxin contamination in the resultant milling fractions.

### 5.2. Byproduct from Bioethanol Production

Conventional bioethanol is produced from corn or wheat either via dry or wet milling. Ethanol corn production process via dry-milling is less capital- and energy-intensive [103]. In the dry grind process, clean corn is ground and mixed with water to form a mash. The mash is cooked and enzymes are added to convert starch to sugar. Then yeasts are added to ferment the sugars, producing a mixture containing ethanol and solids. The solids remaining after distillation are dried to produce byproducts, which can be used as animal feed supplements. The use of ethanol fermentation residues as animal feed is not a new concept but has grown quickly in recent decades. Within the byproducts of bioethanol production, dried distiller’s grains with solubles (DDGS) represent a valuable feed ingredient, particularly replacing expensive protein feed at a competitive price for industry and farmers [104,105,106]. USA, Canada and the EU-28 are the major producers of grain-based ethanol and thereby DDGS. In the USA, ethanol production is based mainly on corn, while in Canada and the EU-28, it is based on both wheat and corn [107]. Worldwide, 6/124 million tons of wheat/coarse grains were used in the manufacture of bioethanol in 2008–2010, increasing to 15/166 million tons in the OECD-FAO forecasts for the year 2020 [107]. The major barriers for an increased acceptance of DDGS as a feed ingredient include the high variability in nutrient composition, making the development of acceptable dietary incorporation rates difficult to assess, and the ever present mycotoxin problem. An extensive review of the chemical composition DDGS has been carried out by Liu [108]. Because nutrient contents in DDGS differ due to several factors, such as raw material origin, processing methods, fermentation yeast properties, and year of production, a complete chemical analysis of each source of DDGS must be performed on a regular basis. An important topic regarding the use of DDGS as animal feed is the associated mycotoxin risk. The occurrence of aflatoxins, DON, fumonisins, T-2 toxin, and ZEA contamination has been reported in DDGS samples from several ethanol plants in the Midwestern United States [16,109]. The level of contamination was very different and generally lower than the advisory levels for use as animal feed provided by the U.S. FDA, with few exceptions. Regarding DON, 12% of the samples that were collected in 2009, a year favorable for DON occurrence in corn, contained DON levels that were higher than the advisory level. No more than 10% of the samples contained fumonisin levels that were higher than recommended for feeding equids and rabbits. A similar picture has been described by Rodrigues and Chin [110]. Corn DDGS samples sourced worldwide were analyzed for aflatoxins, ZEA, DON, fumonisins and OTA. The main result was the high percentage of multi-mycotoxin contamination; 92% of the samples were contaminated with 2 or more mycotoxins. Of the 409 samples that were analyzed, 2%, 8%, 2% and 1% of the DDGS samples exceeded the European feed limits or recommended values for AFB_1_ (20 μg/kg), DON (12,000 μg/kg), fumonisins (5 mg/kg) and ZEA (3,000 μg/kg), respectively. No samples exceeded the threshold levels for OTA. A large survey of mycotoxins in corn DDGS from 78 ethanol plants located in 12 states in the U.S. has been carried out by Khatibi *et al.* [111]. Samples were analyzed for DON, 15-ADON, 3-ADON, NIV, and ZEA. The results were consistent with those previously reported, with a small percentage of DDGS lots containing mycotoxin concentrations above the advisory levels. None of the DDGS lots contained 3-ADON or NIV. Interestingly, a high variability in the mycotoxin contamination of DDGS was evidenced in lots coming from different states. The impact of weather on the mycotoxins in DDGS was not analyzed; however, the authors suggest that the high levels that were observed in the lots from plants located in one state are consistent with reports of increased mycotoxin levels of corn coming from fields that were planted and harvested late and under wet conditions. In samples of wheat based DDGS, the presence of Enniatin B in addition to DON and OTA has been reported [27]. While the available data indicate that mycotoxin contamination in DDGS may represent a low potential health risk, the occurrence, levels, and safety risk of mycotoxins in ethanol byproducts that are used in the feed industry need to be considered and monitored to avoid the exposure of animals to the negative effects of mycotoxin co-contamination. The consequences of the effects of mycotoxins in DDGS on animal health and productivity have modeled to evaluate the economic impact on the livestock industry [106]. In the model, focused on the US context, DDGS contaminated with a single mycotoxin (fumonisin) may contribute to losses in swine production in excess of $147 million annually. Total losses could be significantly higher due to the additive or synergistic effects of mycotoxin co-contamination on animal health.

Monitoring the contamination of raw material and the knowledge of mycotoxin repartitioning during the bioethanol production process are keys factors for the economic viability of fuel-ethanol production to increase the marketability and acceptance of DDGS as feed ingredients. Regarding the variability in DDGS nutrient contents, the level of mycotoxin contamination in DDGS depends on the original grain contamination, processing methods, storage conditions, fermentation yeast properties, and year of production [108]. Although a slight degradation of fumonisins during fermentation has been reported [112], mycotoxins are not destroyed during the ethanol fermentation process or during the production of DDGS. It is generally accepted that some mycotoxins occur in DDGS, representing a potential health and economic risk of ethanol production from corn and a limit for their use in the animal feed industry [106]. An enrichment of DON and ZEA from corn to DDGS of 3–3.5 times has been reported for ethanol industrial plants with different processing parameters [16,113,114]. Unlike the situation for DON, the DON glucoside was not concentrated into DDGS, indicating that some DON glucoside may have been hydrolyzed during the fermentation process and that the ethanol yeasts may hydrolyze the conjugate [114]. An average increase of three times the fumonisin concentration in DDG has been reported by Bowers and Munkvold [112].

Although there are no MLs or guidance levels for grains to be used for industrial purposes, the presence of mycotoxins in grains and their concentration in the byproducts of bioethanol may be a problem for the sustainability of the fuel-ethanol industry. Studies have shown that mycotoxins can affect bioethanol production by stressing fermenting yeasts. ZEA and OTA have the greatest effects in lowering the alcohol productivity, while DON and aflatoxins effects are lower [114,115,116,117,118]. In this context, cereal lots for industrial use should comply with the quality parameter applicable to food and feed. To improve the economic sustainability of the biofuel industry, the surveillance of mycotoxin contamination in grains, kernel cleaning, improved knowledge of mycotoxin repartitioning and fractionation during the ethanol processing, evaluation of alternative bioethanol production process, and monitoring methods represent effective tools to reduce the risk of mycotoxins in DDGS and for a better acceptance of ethanol co-products as feed ingredients [119,120].

### 5.3. Brewing

Beer is the most widely consumed alcoholic beverage, contributing significantly to the diet of the population worldwide. In 2013, a worldwide production of two billion hectoliters was reported [121]. In addition to industrially made beers, consumers demand a wide range of high quality, full-flavored beers from small and independent sources. Craft brewers are meeting this growing demand, creating high levels of economic value in the process. Craft breweries are a vibrant and an important economic force worldwide [122,123]. In this context, knowledge of the effect of malting and beer production process on mycotoxin repartitioning in beer, malt and byproducts is an important topic for human and animal health and the economic sustainability of the barley-malt supply chain. The main use of barley malt is in brewing and in distilling; however, barley malt is now currently used as a component in the food and pharmaceutical products.

The process resulting in the production of beer from barley grain includes the malting and the brewing steps [124]. Malt is made by allowing a grain to germinate, after which it is then dried, ground to a coarse particle size and extracted with hot water. The manufacture of beer involves generation of various residues and byproducts. The most common byproducts are barley rootlets, spent grains, and surplus yeasts. From a feed perspective, considering the use of brewery byproducts as a nutritive component of feedstuffs, the feed industry may play an important role in their revalorization. Barley grains and malt production can be greatly affected by fungal contamination, mainly of *Fusarium* species, with an impact on the safety and quality of malt and beer. Fungal contaminations have in fact been correlated with the beer “gushing” phenomenon, *i.e.*, the over-foaming of beer bottles bursting out the content. An additional effect of fungal infection is the reduction of the quality of produced malt in terms of decreased kernel plumpness and germination [125].

The occurrence of the most common *Fusarium* toxins, such as NIV, DON, deoxynivalenol-3-glucoside (DON-3-Glc), fusarenon-X, 3-ADON, 15-ADON, HT-2 and T-2 toxins and ZEA, has been reported in various barley malting cultivars, with DON representing the most prevalent toxin [17,126,127,128,129,130]. The currently available information on the changes of mycotoxin during barley processing is rather contradictory. Both increases and decreases in DON levels have been observed in malted barley [129]. A reduction of T-2, HT-2 and relevant glucosides from cleaned barley to malt has been observed at rates ranging from 4% to 87% [129,131]. Lancova *et al.* [17] studied the fate of DON, sum of 15- and 3-acetyl18 deoxynivalenol (ADONs), DON-3-Glc, HT-2 toxin, NIV and ZEA during the malting and brewing processes. *Fusarium* mycotoxin accumulation occurred during germination, with a higher content in malt and further increases in beer. The most significant increase was found for DON-3-Glc. These results agree with those reported by Varga *et al.* [130], Kostelanska *et al.* [132], and Schwarz *et al.* [133]. The authors explain this phenomenon as related to the *de novo* growth of *Fusarium* fungi under certain malting conditions or to the release of DON-3-Glc from insoluble forms by enzymes that are produced during the malting process. The prevention of fungal growth in the malting process has been evaluated. Fungicides do not always reduce mycotoxin production in naturally infected cereal grains [134]. Nevertheless, the potential of lactic acid bacteria against spoilage by *Fusarium* fungal contaminants has been demonstrated [135,136]. Lactic acid bacteria in fact can inhibit the growth of molds and thus eliminate the production of associated toxins. In light of this, specific lactic acid bacteria (e.g., *Lactobacillus plantarum*) have been prosed as biopreservative in malting and brewing processes. This potential has been associated to the antifungal activity of certain compounds such as organic acids, as yet uncharacterized proteinaceous compounds, and cyclic dipeptides that can inhibit the growth of some fungi [136]. However, an exhaustive characterization of these antifungal compounds needs further investigations in the near future.

Several byproducts are obtained from the malt and beer industry. Barley rootlets and spent grains represent important feed ingredients due to their high levels of protein and fiber and low price. Mycotoxin exposure resulting from these byproducts should consider not only the raw material characteristics but also the poor management during storage that can result in contamination by mycotoxins. Samples of barley rootlets collected from five maltsters located in Rio de Janeiro State, Brazil, were analyzed for mycotoxins content [137]. All of the samples were positive for FB_1_, whereas AFB_1_ contamination was not detected. Brewer’s spent grain samples from Cordoba Province in Argentina were contaminated with FB_1_, whereas AFB_1_ was found in 18% of brewer’s spent grain, and no detectable levels of AFB_2_, AFG_1_, AFG_2_ or ZEA were reported [138]. There are limited data regarding the repartitioning of mycotoxins from barley to malt byproducts. An enrichment of DON, ADONs, DON-3-Glc, and HT-2 from barley to rootlets has been reported, with values ranging from 5 to 130 times according to the different mycotoxins and the type of contamination, natural *vs.* artificial [128,133]. Enniatins are almost quantitatively transferred to spent grains, probably because of their limited water solubility [91].

In conclusion, the results indicate that mycotoxin contamination in byproducts of the malting and brewing process for animal feeding depends on several factors, including the initial level of barley contamination, the repartitioning of the existing mycotoxins, the variability of the malt and the brewing process technology, the mycotoxin production at various stages of the malting process, the management during storage, *etc.* Knowledge of these factors is still very scarce but is critical for the sustainable use of malt and brewery byproducts in animal nutrition.

## 6. Industrial Use of Cereal Byproducts: Implication for Mycotoxins

As already mentioned, cereal co- and byproducts are important feed raw materials. Byproducts consumption by the feed industry in the EU-28 is about 20 million tons per year [14]. The feed industry is a sustainable outlet for food processing industries, converting byproducts into high-quality animal feed. The FEFAC identified four pillars for a sustainable and competitive feed industry: (1) a safe feed supply; (2) competitive feed and livestock industries; (3) a resource efficient feed industry; and (4) a responsible feed chain [1]. Apart from providing an outlet for co- and byproducts that derive from the production of food and biofuels, the feed sector also offers a sustainable solution for reducing waste further down the production process. The major barriers for an increased acceptance of cereal byproducts as feed ingredients include the high variability in nutrient composition and the ever present mycotoxin problem. However, above these issues, further aspects must be considered in including these products in feed formula, such as economic and marketing issues (formula cost reduction, reduced ingredient market speculation and increased competitiveness) [2]. To properly manage the mycotoxin risk at industrial level, rapid mycotoxin analysis of cereal byproduct represents the first and most effective tool for a better acceptance and use of byproducts as feed ingredients. The main factors affecting mycotoxin contamination of cereal byproduct according to the industrial processing are reported in Table 4.

**Table 4:** Factors affecting mycotoxin contamination in animal feed materials according to the industrial processing of cereal byproducts.

Level in the Industrial Process	Factor
Mycotoxin concentration in (original) cereal grains	Type and level of mycotoxin contamination Cereal processing technology Type of byproducts
Mycotoxin concentration in byproducts	Mycotoxin analysis of byproducts
	Limits/practice in byproducts use

Starting from the contamination levels of byproducts, further actions can be consider to properly manage the mycotoxin risk at the feed industrial level, such as evaluation of the economic value of byproducts and of proper inclusion levels in compound/complete feedstuffs, the use of selected feed additives according to the species sensitivity and carry over potential.

Mycotoxins represent a major analytical challenge due to the wide range of chemical compounds and the wide variety of feed matrices in which they are found. Adequate sampling and analysis are necessary to make justified management decisions regarding what to do with lots that may be contaminated with mycotoxins [55]. Sampling is the critical step to obtain reliable results for the presence of mycotoxins. Sampling is the greatest source of error in quantifying mycotoxin contamination because of the difficulty in obtaining feed samples from large grain consignments and of the uneven distribution of mycotoxins within a commodity [139]. Therefore, planning an effective sampling procedure for cereal mycotoxin detection or quantification represents a complex challenge for operators. The Commission Regulation 401/2006/EC, laying down the methods of sampling and analysis for the official control of the levels of mycotoxins in foodstuffs, provides precise details regarding the methods of sampling, acceptance parameters, criteria for sample preparation, analytical performance criteria of the methods of analysis that are used for the official controls, and criteria for reporting and interpretation of the results [140]. A wide range of analytical methods for mycotoxin determination in food and feed have been developed in recent years, such as high-performance liquid chromatography, gas chromatography, gas chromatography/mass spectrometry and liquid chromatography/mass spectrometry (LC/MS/MS). LC-MS/MS instruments are becoming increasingly widespread for the determination of multiple classes of mycotoxins and of mycotoxin conjugates. Interested readers are referred to the following reviews, which detail the latest developments in mycotoxin analysis [141,142,143,144,145,146,147].

At the feed industry level, the on-site quality of ingredients and finished feeds needs to be continuously monitored. In particular, the areas that require further study and refinement include the conjugated toxin determination and the adoption of a rapid, low-cost, high-throughput analytical approach. From an analytical perspective, the topic of conjugated or modified mycotoxins in feedstuffs, mycotoxin derivatives, that are undetectable by conventional analytical techniques, has been recently considered.

One question is the analytical method of choice for a practical purpose at the industrial level enabling rapid decisions on the acceptance or rejection of a lot. Notwithstanding the availability of advanced methods, the great importance and need for mycotoxin quantification methods at the levels that are set by the European Commission for feedstuffs, the adoption of a rapid, low-cost, high-throughput analytical approach could represent a better option at the industry level, helping to make rapid management decisions [148]. Results from rapid test systems can often be satisfactory as screening methods, while under certain conditions, validated chromatographic methods might be necessary. Commercially available ELISA kits have become very popular due to their relatively low cost and easy application. These kits meet research and industrial needs as “fit-for-purpose”. Fluorescence polarization immunoassays, surface plasmon resonance immunoassays, near- and mid-infrared spectroscopy, and electronic nose represent efficient tools for the detection of fungal and mycotoxin contamination in agricultural commodities [149,150,151,152]. Although rapid methods for on-site mycotoxin measurements are available, the time and effort that are required to obtain a representative sample may still represent a limit for the rapid screening of mycotoxin contamination. Recently, evidence for a significant correlation of concentrations of DON in grain dust and byproducts of grain cleaning with concentrations in whole grains has been given [153,154,155]. Therefore, the sampling and analysis of dust and byproducts of cereal grain cleaning may represent an opportunity to improve on-site rapid mycotoxin measurements and a promising tool for control and mitigate the mycotoxins problem at the industrial level.

## 7. Conclusions and Future Perspective

Mycotoxins represent a significant risk to animal health and are a significant issue for a safe feed supply chain. Despite efforts to control fungal contamination, feed and food mycotoxin contamination is unavoidable and unpredictable. A high incidence of multi-mycotoxin contamination has been reported in feed. Although only a very limited number of mycotoxins have been analyzed, co-contamination is of particular concern due to potential additive or synergistic effects on animals. Regarding this topic, the main future challenges include: (1) increasing the number of analyzed mycotoxins and their metabolites; (2) improving knowledge on the toxicological effects of co-occurring mycotoxins; and (3) risk assessment regarding the carry-over of mycotoxins and metabolites that are usually considered negligible. These data are critical to revise guidelines for maximum levels in feed to ensure maximum protection for animal and human health.

The world population is increasing rapidly. There is an increasing competition between humans and animals for food commodities, and an increasing proportion of edible grain is being diverted to biofuel production. These different markets may be supplied by different grade of grains with different costs. Therefore, there will be a greater availability of food byproducts as animal feed sources, which may also be of a lower quality. The feed industry is a sustainable outlet for the food processing industries, converting byproducts into high-quality animal feed. Moreover, the marketability and suitable uses of food process byproducts are keys to the economic viability of food and biofuel production. Studies on the fate of mycotoxins during food processing have shown that mycotoxins are concentrated into fractions that are commonly used as animal feed. A high variability in mycotoxin contamination of cereal byproducts has been evidenced, representing barriers for an increased acceptance of several food byproducts as feed ingredients. Several factors affect the mycotoxin repartitioning in food process byproducts, such as the initial level of cereal contamination, kernel localization, and the high variability of the different food process technologies.

A precise understanding of how mycotoxin distribution and concentration change during the technological processes is critical. Knowledge of mycotoxin repartitioning in cereal milling fractions is more widespread, although it is still largely limited to DON. Data regarding mycotoxin repartitioning in byproducts from the bioethanol and brewery industries are still limited, requiring a better knowledge of the industrial processing technology. Future attention should be paid not only to the mycotoxins that are believed to be the most likely to occur but also to the presence of mycotoxin co-contamination and modified mycotoxins. The additive/synergistic effects of mycotoxins on animal health still need to be evaluated. Science-based information concerning these topics is needed to use food byproducts efficiently, effectively, safely and profitably in the feed supply chain. Moreover, these data may support risk management and regulatory bodies to reduce human and animal exposure to dangerous amounts of mycotoxins and to revise legislative limits.

The high variability in mycotoxin contamination of cereal byproducts begs increased awareness and ongoing surveillance for mycotoxins. Regular, economical and straightforward feed sampling and testing with regard to a rapid and accurate diagnosis of feed quality are needed. The development of rapid methods for use in the field represents a future challenge, but such methods would allow for “decision-making” regarding the safe use of a given feed in animal feeding. However, more research on the development and application of multi-mycotoxin analytical methods should be encouraged in order to obtain a more accurate picture of the extent of multi-mycotoxin contamination.

The impact of mycotoxins entering the feed supply chain could increase in the future. Most predictions indicate that climate change scenarios, with global warming, could affect agriculture and increase and change the threat of fungal invasion of crops.
